# The reliability of back-extrapolation in estimating ${\dot{V}O}_{2peak}$ in different swimming performances at the severe-intensity domain

**DOI:** 10.3389/fphys.2022.982638

**Published:** 2022-11-03

**Authors:** Danilo Alexandre Massini, Astor Reis Simionato, Tiago André Freire Almeida, Anderson Geremias Macedo, Mário Cunha Espada, Joana Filipa Reis, Francisco Besone Alves, Dalton Müller Pessôa Filho

**Affiliations:** ^1^ Postgraduate Program in Human Development and Technologies, São Paulo State University—UNESP, Rio Claro, São Paulo, Brazil; ^2^ São Paulo State University—UNESP, Bauru, São Paulo, Brazil; ^3^ CIPER, Faculdade de Motricidade Humana, University de Lisboa, Lisboa, Portugal; ^4^ Polytechnic Institute of Setúbal (CIEF—ESE/IPS, CDP2T, ESTSetúbal/IPS), Setúbal, Portugal; ^5^ Life Quality Research Centre (CIEQV-Leiria), Rio Maior, Portugal; ^6^ Faculdade de Motricidade Humana, Universidade de Lisboa, Lisboa, Portugal; ^7^ Portugal Football School, Portuguese Football Federation, FPF, Cruz Quebrada, Portugal

**Keywords:** swimming, back-extrapolation, peak oxygen uptake, oxygen uptake kinetics, oxygen uptake recovery

## Abstract

The amount of anerobic energy released during exercise might modify the initial phase of oxygen recovery (fast-O_2debt_) post-exercise. Therefore, the present study aimed to analyze the reliability of peak oxygen uptake $\left({\dot{V}O}_{2peak}\right)$ estimate by back-extrapolation $\left(BE-{\dot{V}O}_{2peak}\right)$ under different swimming conditions in the severe-intensity domain, verifying how the alterations of the ${\dot{V}O}_{2}$ recovery profile and anerobic energy demand might affect $BE-{\dot{V}O}_{2peak}$ values. Twenty swimmers (16.7 ± 2.4 years, 173.5 ± 10.2 cm, and 66.4 ± 10.6 kg) performed an incremental intermittent step protocol (IIST: 6 × 250 plus 1 × 200 m, IIST_v200m) for the assessment of ${\dot{V}O}_{2peak}$ . The ${\dot{V}O}_{2}$ off-kinetics used a bi-exponential model to discriminate primary amplitude, time delay, and time constant (A_1off_, TD_1off_, and τ_off_) for assessment of fast-O_2debt_ post IIST_v200m, 200-m single-trial (v200 m), and rest-to-work transition at 90% delta (v90%Δ) tests. The linear regression estimated $BE-{\dot{V}O}_{2peak}$ and the rate of ${\dot{V}O}_{2}$ recovery (BE-slope) post each swimming performance. The ANOVA (Sidak as *post hoc*) compared ${\dot{V}O}_{2peak}$ to the estimates of $BE-{\dot{V}O}_{2peak}$ in v200 m, IIST_v200 m, and v90%Δ, and the coefficient of dispersion (R^2^) analyzed the association between tests. The values of ${\dot{V}O}_{2peak}$ during IIST did not differ from $BE-{\dot{V}O}_{2peak}$ in v200 m, IIST_v200 m, and v90%Δ (55.7 ± 7.1 vs. 53.7 ± 8.2 vs. 56.3 ± 8.2 vs. 54.1 ± 9.1 ml kg^−1^ min^−1^, *p* > 0.05, respectively). However, the ${\dot{V}O}_{2peak}$ variance is moderately explained by $BE-{\dot{V}O}_{2peak}$ only in IIST_v200 m and v90%Δ (R_Adj_^2^ = 0.44 and R_Adj_^2^ = 0.43, *p* < 0.01). The TD_1off_ and τ_off_ responses post IIST_v200 m were considerably lower than those in both v200 m (6.1 ± 3.8 and 33.0 ± 9.5 s vs. 10.9 ± 3.5 and 47.7 ± 7.9 s; *p* < 0.05) and v90%Δ ( 10.1 ± 3.8 and 44.3 ± 6.3 s, *p* < 0.05). The BE-slope post IIST_v200m was faster than in v200 m and v90%Δ (-47.9 ± 14.6 vs. -33.0 ± 10.4 vs. -33.6 ± 13.8 ml kg^−1^, *p* < 0.01), and the total anerobic (Anaer_Total_) demand was lower in IIST_v200 m (37.4 ± 9.4 ml kg^−1^) than in 200 m and 90%Δ (51.4 ± 9.4 and 46.2 ± 7.7 ml kg^−1^, *p* < 0.01). Finally, the τ_1off_ was related to Anaer_Total_ in IIST_v200m, v200 m, and v90%Δ (r = 0.64, r = 0.61, and r = 0.64, *p* < 0.01). The initial phase of the ${\dot{V}O}_{2}$ recovery profile provided different (although reliable) conditions for the estimate of ${\dot{V}O}_{2peak}$ with BE procedures, which accounted for the moderate effect of anerobic release on ${\dot{V}O}_{2}$ off-kinetics, but compromised exceptionally the ${\dot{V}O}_{2peak}$ estimate in the 200-m single trial.

## Introduction

Back-extrapolation (BE) has been demonstrated to be a suitable procedure for estimating the peak oxygen uptake $\left({\dot{V}O}_{2peak}\right)$ at the very end of exercise by applying the linear ${\dot{V}O}_{2}$ -time relationship to the primary response of the ${\dot{V}O}_{2}$ recovery phase (i.e., fast ${\dot{V}O}_{2}$ off-kinetics) (Léger et al., 1980; Rodríguez et al., 2017; Monteiro et al., 2020). In swimming, BE is a reliable procedure for estimating ${\dot{V}O}_{2peak}$ attained in an incremental exercise (Lavoie et al., 1981; Montpetit et al., 1981), and even BE affords a reliable estimate of ${\dot{V}O}_{2peak}$ during middle-distance swimming performances (i.e., 200 and 400 m), in which the attainment of the maximal rate of aerobic energy is recognized (Chaverri et al., 2016; Rodríguez et al., 2017). Therefore, the ${\dot{V}O}_{2peak}$ estimate from BE is supposed to provide the assessment of maximum ${\dot{V}O}_{2}$ response from submaximal to supramaximal swimming circumstances (Monteiro et al., 2020), and thus BE is also considered a procedure enabling the overcome of contextual constraints imposed by the apparatus for the assessment of ${\dot{V}O}_{2}$ response in the aquatic environment (Chaverri et al., 2016).

However, the linear ${\dot{V}O}_{2}$ -time model has been the source of controversial findings on the reliability of BE to estimate ${\dot{V}O}_{2peak}$ in swimming (Lavoie et al., 1985; Chaverri et al., 2016). For example, the overestimation of ${\dot{V}O}_{2peak}$ assessment of a post 400-m single-trial swimming performance (Lavoie et al., 1981) conflicts with the post incremental step-test values (Montpetit et al., 1981), despite both being swimming circumstances with a recognized maximum ${\dot{V}O}_{2}$ demand (Zacca et al., 2019). Probably, this mismatch in comparing BE estimate vs. incremental test assessment of ${\dot{V}O}_{2peak}$ might account for the impairments on physiological response during high-intensity constant work-rate exercise, including either oxidative inertia or the anerobic energy relying on the onset of exercise since both these physiological mechanisms are supposed to modulate ${\dot{V}O}_{2}$ off-kinetics acutely (i.e., slowing or speeding ${\dot{V}O}_{2}$ exponential response post-exercise) (Özyener et al., 2001; Rossiter et al., 2002; Sousa et al., 2015). However, these physiological mechanisms are assumed to impair the attainment of ${\dot{V}O}_{2peak}$ during constant-phase exercise, if the reference value for comparison (usually assessed from an incremental exercise protocol) might be considered a reliable ${\dot{V}O}_{2peak}$ in swimming (Sousa et al., 2014; Pessôa Filho et al., 2017).

Despite the factors influencing BE reliability to estimate ${\dot{V}O}_{2peak}$ , previous reports suggested both the 200- and 400-m performances in swimming as typical middle-distance events, eliciting high aerobic energy release and, therefore, the attainment of ${\dot{V}O}_{2peak}$ response, in spite of the differences between each other regarding the aerobic/anerobic energetics balance (Pyne and Sharp, 2014; Almeida et al., 2020; Zacca et al., 2020). In addition, it has been demonstrated that velocities between 95 and 105% of ${\dot{V}O}_{2peak}$ in swimming also elicited the ${\dot{V}O}_{2peak}$ (Sousa et al., 2014) and showed a similar profile of ${\dot{V}O}_{2}$ response when compared to 200- and 400-m performance (Sousa et al., 2011; Chaverri et al., 2016; Rodríguez et al., 2017). Therefore, the 200- and 400-m trials might be considered suitable for estimating ${\dot{V}O}_{2peak}$ by applying BE procedures post all-out performances in swimming (Rodríguez et al., 2017; Zacca et al., 2019).

From these studies, the main lessons are that the BE procedure might overestimate the ${\dot{V}O}_{2peak}$ according to the dataset fitting strategies, the exercise intensity during a trial performance (Rodríguez et al., 2017), and exercising conditions previous to the target trial estimating ${\dot{V}O}_{2peak}$ (Rodríguez et al., 2017; Zacca et al., 2019). In other words, the mechanisms that affect the reliability of the ${\dot{V}O}_{2peak}$ estimate by BE are likely related to the physiological response during exercise that also affects the ${\dot{V}O}_{2}$ kinetic responses in the recovery phase. This is if other sources capable of impairing the accuracy of the BE estimate (e.g., temporal resolution of data sampling, treatment of the dataset, and mathematical curve fitting) are dis-regarded. (for further information on these other sources, see Monteiro et al., 2020; Rodríguez et al., 2017). Such a relationship was theoretically supposed to explain the modification of the constants of the linear function with the increase of the delay for the onset of ${\dot{V}O}_{2}$ recovery, which was in turn, linked to the velocity of ${\dot{V}O}_{2}$ adjustment during exercise (i.e., ${\dot{V}O}_{2}$ on-kinetic) (Rodríguez et al., 2017).

In fact, experimental results have postulated that a high and rapid increase of ${\dot{V}O}_{2}$ during exercise is related to a similar high and rapid reduction in the muscle phosphocreatine (PCr) content, the restoration of which inhibits the rapid decline of oxidative phosphorylation in the initial phase of recovery after exercise (i.e., slow time constant of ${\dot{V}O}_{2}$ off-kinetic—τ_off_) (Rossiter et al., 2002; Korzeniewski and Zoladz, 2013). Indeed, this assumption might also support the overestimation of ${\dot{V}O}_{2peak}$ when applying BE procedures post 400 m rather than post 200 m (Rodríguez et al., 2017). Despite not ever being addressed, the τ_off_ might play an important role for explaining how the reliability of BE to estimate ${\dot{V}O}_{2peak}$ is affected by performing exercises in different circumstances, leading to the attainment of the maximal aerobic rate.

Thus, the current study aimed to address the ${\dot{V}O}_{2}$ recovery response and anerobic energy demand post different swimming circumstances in the severe-intensity domain to ascertain whether transients of ${\dot{V}O}_{2}$ off-kinetics account for alterations of the linear adjustments of ${\dot{V}O}_{2}$ response during the initial phase of ${\dot{V}O}_{2}$ off-kinetics. Hence, the gathering of information to analyze the reliability of BE in estimating ${\dot{V}O}_{2peak}$ values with correspondence to the maximal ${\dot{V}O}_{2}$ elicited whatever the swimming demand upon anerobic energetics during performances in the severe-intensity domain and correspondence to the maximum ${\dot{V}O}_{2}$ response assessed in incremental exercise. In addition, this study explored whether a 200-m single-trial performance would be a feasible reference for the estimation of ${\dot{V}O}_{2peak}$ , adding information to support (or not) that the value estimated by BE is similar to either the ${\dot{V}O}_{2peak}$ assessed in an incremental test and/or the maximal ${\dot{V}O}_{2}$ elicited at the end of the trial.

## Methods

### Subjects

Twenty swimmers (16.7 ± 2.4 years, 173.5 ± 10.2 cm, and 66.4 ± 10.6 kg; men = 12 and women = 8) were voluntarily recruited to participate in the study. The swimmers had at least three annual competitive training seasons and 200-m performances corresponding to 533 ± 83 and 502 ± 75 FINA points in a 25-m swimming pool, respectively, for men and women. The experimental procedures were performed in an indoor 25-m swimming pool, with a water temperature of ∼28°C. The swimmers were evaluated after familiarization with the procedures and devices. They were instructed to refrain from exhaustive training, alcohol, and caffeinated drinks the day before testing and to arrive well-fed and hydrated for the tests. All swimmers (and their legal guardians when they were under 18 years of age) signed a written consent form for their participation. This research was approved by the local ethics committee (CAEE: 54372516.3.0000.5398).

### Performance tests and incremental intermittent step test (IIST)

**FIGURE 1:**
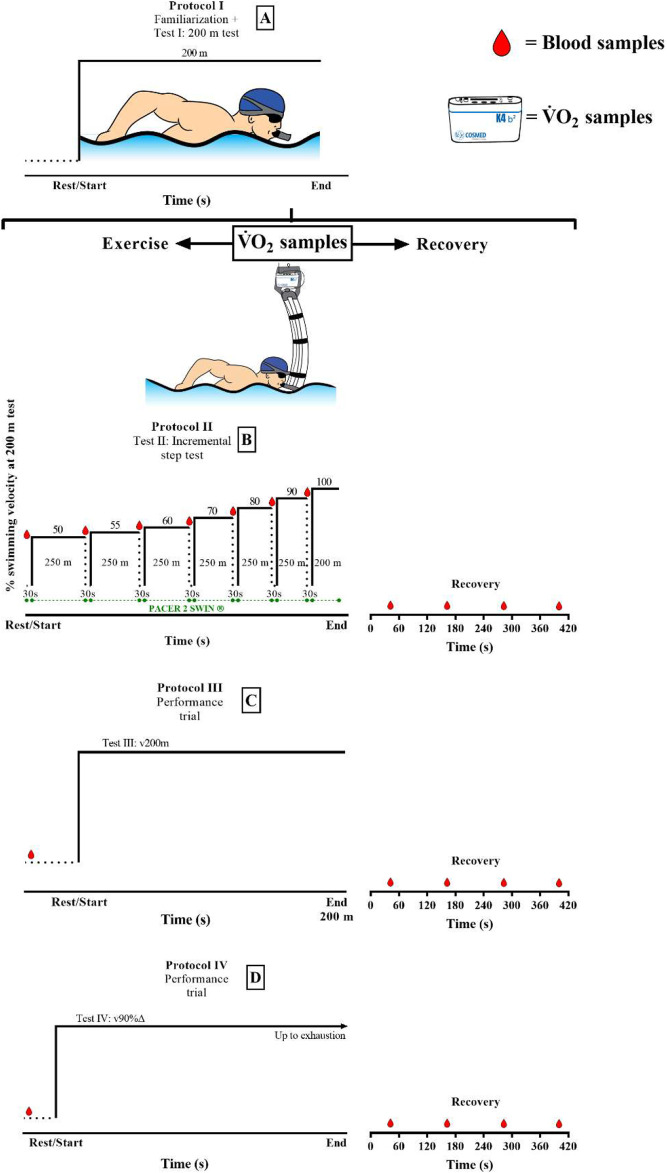
Illustration of the protocols: **(A)** familiarization with snorkel and 200-m single-test trial both with no gas sampling; **(B)** incremental intermittent test including 200-m last-step performance (IIST_200m); **(C)** single-trial performance during 200 m (v200m) and **(D)** rest-to-work transition to the limit of tolerance at delta 90% velocity (v90%Δ).

The familiarization phase with the snorkel system took place 24 h before testing procedures, which included all components of a regular training session, emphasizing middle-distance conditioning. All swimmers performed three swimming tests, with the duration between them being at least 48 h (Figure 1), with the second and third tests performed in a randomized order. The tests were 1) an incremental intermittent step-test (IIST) composed of six sets of 250 m in addition to one set of 200 m (IIST_v200m) at 50, 55, 60, 70, 80, 90, and 100% of velocity for 200 m, with 30 s between each step for blood sampling analysis (Almeida et al., 2021). The 200-m test was performed just after familiarization had been accomplished and 24 h before the IIST, following: 1) 1 h of rest from the previous exercise bout and 2) executed maximally with water starting, open turns, and no underwater gliding, as suggested by Massini et al. (2021); 2) a maximal 200-m single-trial performance (v200m); and 3) a transition from rest to the velocity corresponding to 90%Δ (v90%Δ, Eqn. (1)) performed until volitional exhaustion.

$$v90\%∆=v_{LT}+[\left({v\dot{V}O}_{2\max}-v_{LT}\right)\times 0.9],$$ [1]

where v_LT_ is the velocity corresponding to the lactate threshold (LT), defined as the first increase of blood lactate concentration ([la^−^]) above the resting levels, and determined from log–log bi-segmented plots of [la^−^] vs. velocity during the IIST (Faude et al., 2009). The swimming speed during all tests was controlled by visual information using an underwater visual pacer placed along the bottom of the pool (Pacer2Swim^®^, KulzerTEC, Portugal).

## Measurements

Breath-by-breath gas exchange was sampled during and after the following experimental conditions: IIST, v200 m, and v90%Δ. For all conditions, the portable CPET unit (K4b^2^, Cosmed, Italy) was attached to the swimmer by a specific snorkel (new-AquaTrainer^®^, Cosmed, Italy), which was validated for gas analysis in swimming by Baldari et al. (2013). The CPET unit was calibrated before each test following the manufacturer’s recommendations. Blood samples (25 ul) were obtained from the swimmers’ earlobe at rest and at 1, 3, 5, and 7 min post-exercise, which were diluted in 75 ul 1% NaF solution. The samples were immediately analyzed for [la^−^] evaluation (YSI, 2300 STAT, Yellow Springs, United States).

For assessment of ${\dot{V}O}_{2peak}$ and peak aerobic velocity $\left({v\dot{V}O}_{2peak}\right)$ during the IIST, the ${\dot{V}O}_{2}$ data were smoothed (3-data point filter) and time-aligned to the discernibility of exercise and recovery phases. Moving average (30 s) processing was applied to the exercise ${\dot{V}O}_{2}$ raw data, and the highest averaged value was considered the ${\dot{V}O}_{2peak}$ (Robergs et al., 2010; Reis et al., 2012). The velocity corresponding to the step of ${\dot{V}O}_{2peak}$ occurrence was defined as ${v\dot{V}O}_{2peak}$ . For modeling of ${\dot{V}O}_{2}$ off-kinetics, the 420-s rough ${\dot{V}O}_{2}$ dataset from each transition at v200 m, v90%Δ, and IIST_v200m was time-aligned, and the noise was excluded and interpolated second-to-second for the analysis of ${\dot{V}O}_{2}$ off-kinetics, as suggested by Özyener et al. (2001), Keir et al. (2014), and Benson et al. (2017). The mathematical modeling of ${\dot{V}O}_{2}$ off-kinetics used a bi-exponential equation, with time delay (TD) (Eqn. (2)), according to the recommendations of Özyener et al. (2001) for the modeling of ${\dot{V}O}_{2}$ off-kinetics in severe exercise:

$$\dot{V}O_{2off}\left(t\right)=EE\dot{V}O_{2}-A_{1off}\left[1-e^{-\left({t-TD}_{1off}/\tau_{1off}\right)}\right]-A_{2off}\left[{1-e}^{-\left({t-TD}_{2off}/\tau_{2off}\right)}\right]$$ [2]

where $EE{\dot{V}O}_{2}$ corresponded to the final 30 s averaged ${\dot{V}O}_{2}$ increase during exercise (in ml min^−1^). A_1off_ and A_2off_ are the net amplitude of ${\dot{V}O}_{2}$ response for each phase of recovery (in ml∙min^−1^); t is exercise time; τ_1off_ and τ_2off_ are time constants (in seconds, s); and TD_1off_ and TD_2off_ are the time delays (in seconds, s) for ${\dot{V}O}_{2}$ response for each phase of recovery (Özyener et al., 2001). The cardiopulmonary component was excluded by adjusting ${\dot{V}O}_{2}$ response ∼15 s after the onset of exercise recovery (Özyener et al., 2001). The fast-O_2debt_ (i.e., the amount of ${\dot{V}O}_{2}$ response up to a particular time of the initial ${\dot{V}O}_{2}$ recovery phase) was calculated from Eqn. (3), as recommended by Stirling et al. (2005):

Fast−O2debt=A1off·τ1off(1−e(tf−TD1off)τ1off)+A1off×(TD1off−tf)e(tf−TD1off)τ1off, [3]

where t_f_ is the time (s) at the end of the recovery sampling protocol. The blood lactate accumulation in equivalents of O_2_ (O_2_[la^−^], in ml∙min^−1^) was calculated following the recommendations of Prampero and Ferretti (1999) from O_2_[la^−^] = β·[la^−^]_net_, where β is equivalent to 2.7 ml kg^−1^ per 1 mmol L^−1^ of [la^−^]_net_, which is the algebraic difference between rest [la^−^] and peak [la^−^] post-exercise. The fast-O_2debt_ (in ml·kg^−1^) and O_2_[la^−^] variables indicated the phosphagen and glycolytic components of total anerobic (Anaer_Total_) response, respectively, during each swimming performance trial. The mean response time for the fast-O_2_debt curve was calculated (MRT_1off_ = TD_1off_ + τ_1off_, s) according to the previous studies in swimming (Almeida et al., 2020; Massini et al., 2021).

The BE method was applied to estimate the ${\dot{V}O}_{2peak}$ ( $BE-{\dot{V}O}_{2peak}$ , in ml min^−1^) and ${\dot{V}O}_{2}$ recovery rate (BE-slope, in ml kg^−1^) from post-exercise ${\dot{V}O}_{2}$ response (Montpetit et al., 1981) in IIST_v200m, v200 m, and v90%Δ. This procedure adjusted 20 s of the ${\dot{V}O}_{2}$ vs. recovery time dataset by a linear function (f(y) = ax + b) (Léger et al., 1980), in which the delay of ${\dot{V}O}_{2}$ recovery response (i.e., ∼15 s) was excluded before the linear adjustment of the dataset (see details on cardiopulmonary component exclusion for mathematical modeling of ${\dot{V}O}_{2}$ off-kinetics) to the zero-recovery time.

### Statistical analysis

The ${\dot{V}O}_{2peak}$ , $EE{\dot{V}O}_{2}$ , and $BE-{\dot{V}O}_{2peak}$ values (in ml·kg^−1^ min^−1^) for each trial were checked for normality with the Shapiro–Wilk test. The one-way ANOVA (Sidak as *post hoc*) compared ${\dot{V}O}_{2peak}$ to $BE-{\dot{V}O}_{2peak}$ and $EE{\dot{V}O}_{2}$ in the IIST_v200m, v200m, and v90%Δ and the values of τ_1off_, TD_1off_, MRT_1off_, A_1off_, $EE{\dot{V}O}_{2}$ , fast-O_2debt_, BE-Slope, and O_2_[la^−^] between each of the swimming performance conditions. The coefficient of dispersion (R^2^) and standard error of estimate (SEE) analyzed the variance between ${\dot{V}O}_{2peak}$ and $BE-{\dot{V}O}_{2peak}$ . Eta squared (η^2^) was calculated to determine the effect size for ANOVA, considering the threshold values as <0.04 [trivial], 0.04–0.24 [small], 0.25–0.63 [medium], and >0.64 [large] (Fergusson, 2009).

Pearson’s coefficient (r) analyzed the correlation of ${\dot{V}O}_{2}$ off-transients, fast-O_2debt_, and O_2_[la^−^] with $EE{\dot{V}O}_{2}$ , $BE-{\dot{V}O}_{2peak}$ , BE-slope, and ${\dot{V}O}_{2}$ off-kinetic components under each swimming condition. The magnitudes of Pearson’s correlation were expressed as weak (0.00–0.29), low (0.30–0.49), moderate (0.50–0.69), strong (0.70–0.89), or very strong (0.90–1.00) (Mukaka, 2012); while R^2^ was considered <0.04 [trivial], 0.04–0.24 [small], 0.25–0.63 [medium], and >0.64 [strong] (Fergusson, 2009). For all analyses, the significance level was set at ρ ≤ 0.05. Sample power for the observed correlations was calculated considering the sample size (*n* = 20), correlation coefficient (r) Zα = 1.96 to a security index of α = 0.05, and expected sample power of 80% (β = 0.20). The statistical analysis was performed with SPSS Statistics for Windows (v18.0, IBM^®^, Chicago, IL, United States), and ${\dot{V}O}_{2}$ data processing and modeling were both performed using OriginPro (OriginLab Corporation^®^, Northampton, MA, United States).

## Results

**FIGURE 2:**
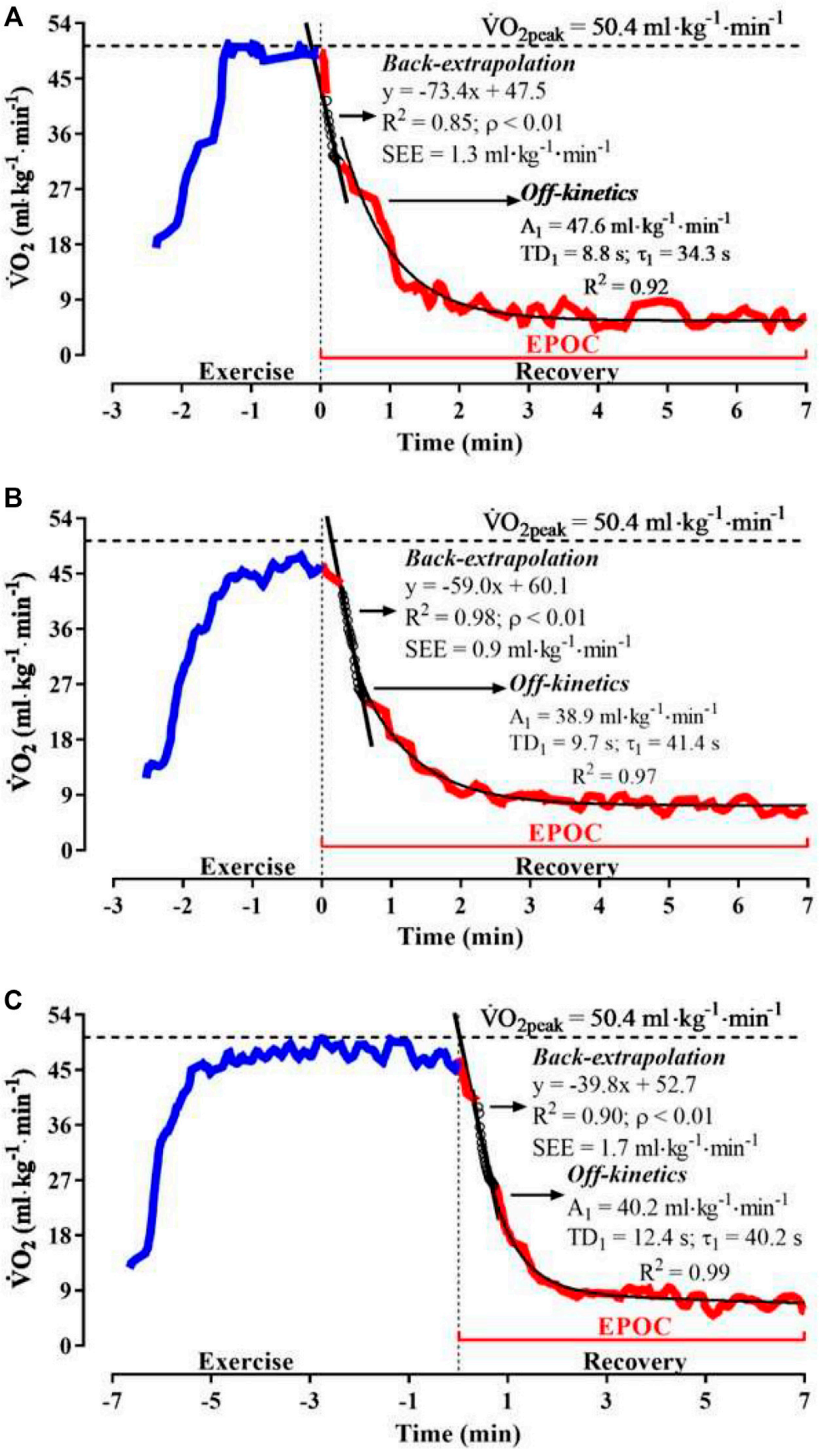
Illustration of the procedures applied to adjust recovery ${\dot{V}O}_{2}$ “on” (blue) and “off” (red) profiles during IIST_200m **(A)**, v200m **(B)**, and v90%Δ **(C)** for the subject #7.

The ${\dot{V}O}_{2peak}$ attained in the IIST was 55.7 ± 7.1 ml·kg^−1^·min^−1^, and $v{\dot{V}O}_{2peak}$ corresponded to 1.26 ± 0.08 m × s^−1^. The v90%Δ and v200m were performed at 96.3 ± 4.4 and 101.1 ± 5.1% of $v{\dot{V}O}_{2peak}$ , respectively Figure 2 illustrates the ${\dot{V}O}_{2}$ response profile during exercise and recovery of IIST_v200m, v200 m, and v90%Δ for a male swimmer, which also exemplifies the “off-kinetics” and linear “back-extrapolation” modeling.

The variables of ${\dot{V}O}_{2}$ off-kinetics and BE are shown in Table 1. Differences were observed for TD_1off_, τ_1off_, and MRT_1off_ (*p* < 0.01, η^2^= 0.251, 0.397, and 0.479, all considered [medium] effect size), which were lower in IIST_v200m than in v200 m and v90%Δ, but not between v200 m and v90%Δ (ρ = 0.84, 0.45, and 0.35). No differences were observed for A_1off_ (F_[2,57]_ = 0.18, *p* = 0.83, η^2^= 0.006 [trivial]) and $EE{\dot{V}O}_{2}$ (F_[2,57]_ = 0.04, *p* = 0.96, η^2^= 0.001 [trivial]) between trials.

**TABLE 1:** Mean ± SD values for ${\dot{V}O}_{2}$ off-transients and constants of BE in IIST_200m, v200 m, and v90%Δ. Measurements of the goodness and variability for linear fitting are also shown. N = 20.

	IIST_v200m	v200m	v90%Δ
${\dot{V}O}_{2}$ ** off-kinetics**			
TD_1off_ (s)	6.1 ± 3.8	10.9 ± 3.5*	10.1 ± 3.8*
τ_1off_ (s)	33.0 ± 9.5	47.7 ± 7.9*	44.3 ± 6.3*
MRT_1off_ (s)	39.1 ± 10.8	58.7 ± 8.3**	54.3 ± 7.6**
A_1off_ (ml·kg^−1^·min^−1^)	44.0 ± 8.5	45.0 ± 6.8	45.3 ± 6.3
$EE{\dot{V}O}_{2}$ (ml·kg^−1^·min^−1^)	53.7 ± 7.0	53.2 ± 6.9	53.5 ± 6.3
% ${\dot{V}O}_{2peak}$	96.5 ± 3.5	96.2 ± 12.4	96.5 ± 7.4
R^2^	0.96 ± 0.03	0.98 ± 0.01	0.98 ± 0.01
**Linear coefficients**			
$BE-{\dot{V}O}_{2peak}$ (ml·kg^−1^·min^−1^)	53.7 ± 8.9	56.3 ± 8.3	54.1 ± 9.1
SEM (ml·kg^−1^·min^−1^)	2.0	1.9	2.0
BE-slope (ml·kg^−1^)	-47.9 ± 14.6	-33.0 ± 10.4*	-33.6 ± 13.8*
SEM (ml·kg^−1^)	3.3	2.3	3.1
% ${\dot{V}O}_{2peak}$	96.6 ± 11.5	101.7 ± 14.3	96.5 ± 7.4
R^2^	0.91 ± 0.08	0.95 ± 0.04	0.96 ± 0.04

(*) significantly different from IIST_200m at ρ ≤ 0.05. (**) significantly different from IIST_200m at ρ ≤ 0.01. SEM: standard error of mean.

In addition, $BE-{\dot{V}O}_{2peak}$ values did not differ between trials (*p* = 0.62), despite BE-slope being higher (*p* < 0.01, η^2^= 0.227, considered [small] effect size) in the IIST_v200m than in the v200m and v90%Δ (*p* < 0.01 for both comparisons), but no difference was observed between v200m and v90%Δ (ρ = 1.00). The values of $BE-{\dot{V}O}_{2peak}$ assessed for IIST_v200m, v200m, and v90%Δ (Table 1) were not different from those of ${\dot{V}O}_{2peak}$ (*p* = 0.73), neither were differences observed when comparing the EE $\dot{V}$ O_2_ during each trial for $BE-{\dot{V}O}_{2peak}$ (*p* = 0.84) or ${\dot{V}O}_{2peak}$ (*p* = 0.65).

**FIGURE 3:**
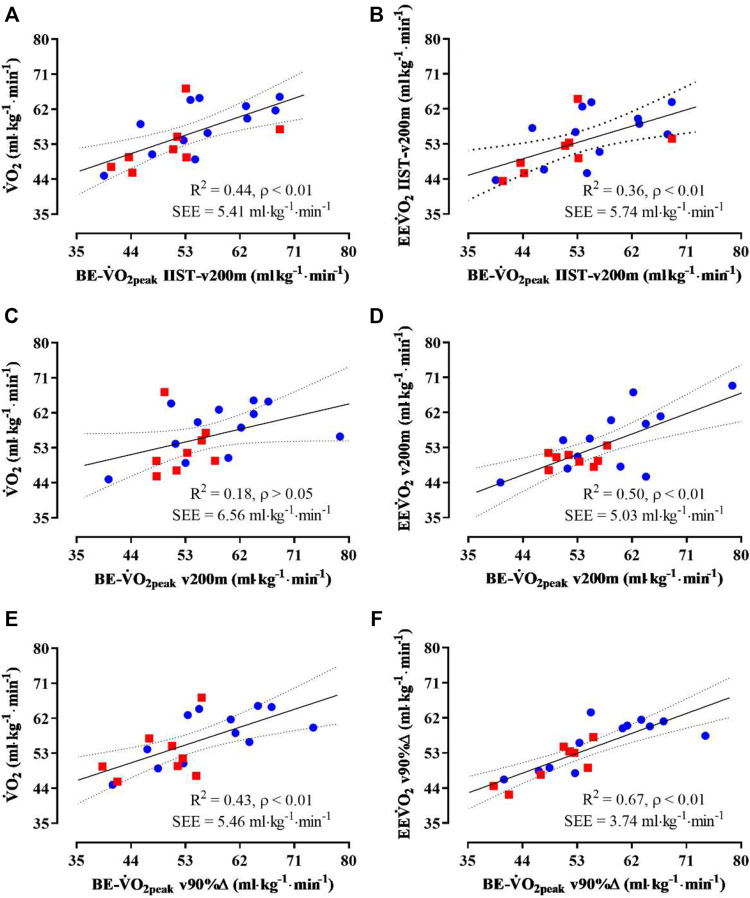
Linear regression analysis between the values of VO_2max_ and $BE-{\dot{V}O}_{2peak}$ for IIST_v200m **(A)**, v200m **(B)**, and v90%Δ **(C)** and between $EE{\dot{V}O}_{2}$ and $BE-{\dot{V}O}_{2peak}$ for IIST_v200m **(D)**, v200m **(E)**, and v90%Δ **(F)**. Red-filled square: women (N = 8) and blue-filled circle: men (N = 12). SEE: standard error of estimate.

Small-to-medium R^2^ coefficients were observed between ${\dot{V}O}_{2peak}$ and $BE-{\dot{V}O}_{2peak}$ for IIST_v200m, v200 m, and v90%Δ (Figure 3, panels A, C, and E, respectively), but a non-significant R^2^ coefficient was observed between ${\dot{V}O}_{2peak}$ and $BE-{\dot{V}O}_{2peak}$ for v200 m. Also, the R^2^ coefficients were medium to strong between EE $\dot{V}$ O_2_ and $BE-{\dot{V}O}_{2peak}$ for IIST_v200m, v200m, and v90%Δ (Figure 3, panels B, D, and F, respectively).

Pearson’s coefficients between parameters of both models (i.e., ${\dot{V}O}_{2}$ off-kinetics vs. BE) attained satisfactory sample power and showed moderate-to-strong correlations between A_1off_ with $BE-{\dot{V}O}_{2peak}$ and BE-slope for the IIST_v200m and v90%Δ trials, whereas for the v200 m trial, these correlations ranged from low to moderate (Table 2).

**TABLE 2:** Pearson’s coefficients between the variables of ${\dot{V}O}_{2}$ off-kinetics with $EE{\dot{V}O}_{2}$ , $BE-{\dot{V}O}_{2peak}$ , and BE-Slope for IIST_200m, v200 m, and v90%Δ. N = 20.

	${\dot{V}O}_{2}$ off-kinetics			
	**TD_1off_ (s)**	**τ_1off_ (s)**	**MRT (s)**	**A_1off_ (ml ⋅ kg^−1^ ⋅ min^−1^)**
**IIST_v200m**
$EE{\dot{V}O}_{2}$ (ml·kg^−1^·min^−1^)	ns	ns	ns	0.74**
$BE-{\dot{V}O}_{2peak}$ (ml·kg^−1^·min^−1^)	ns	ns	ns	0.55*
BE-slope (ml·kg^−1^)	ns	ns	ns	ns
**v200m**
$EE{\dot{V}O}_{2}$ (ml·kg^−1^·min^−1^)	ns	ns	ns	0.67**
$BE-{\dot{V}O}_{2peak}$ (ml·kg^−1^·min^−1^)	ns	ns	ns	0.48*
BE-slope (ml·kg^−1^)	ns	-0.45*	-0.44*	ns
**v90%Δ**
$EE{\dot{V}O}_{2}$ (ml·kg^−1^·min^−1^)	ns	ns	ns	0.82**
$BE-{\dot{V}O}_{2peak}$ (ml·kg^−1^·min^−1^)	ns	ns	ns	0.83**
BE-slope (ml·kg^−1^)	ns	ns*	-0.47*	ns

(*) coefficient with significance at ρ ≤ 0.05; (**) coefficient with significance at ρ ≤ 0.01; (*ns*) coefficient with no significance.

**FIGURE 4:**
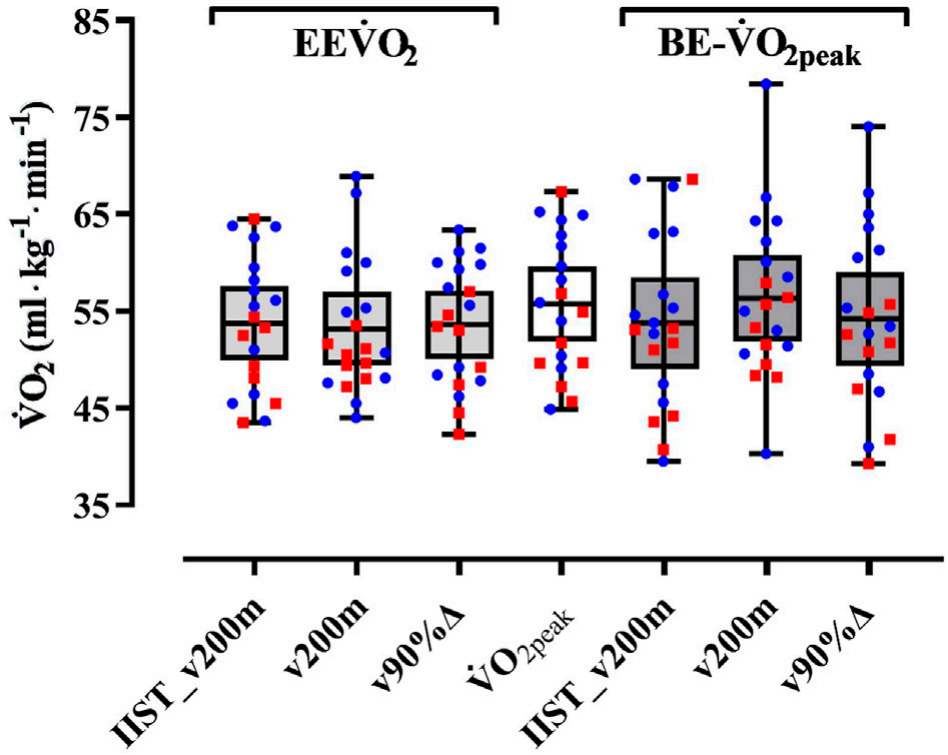
Box plots illustrating the variability of maximal and peak ${\dot{V}O}_{2}$ measurements ( $EE{\dot{V}O}_{2}$ and ${\dot{V}O}_{2peak}$ ) and estimates ( $BE-{\dot{V}O}_{2peak}$ ) during each trial (IIST_v200m, v200m, and v90%Δ). The central horizontal line inside squares depicts the mean values, the bottom and top lines of squares indicate the lower and upper boundaries for 95% confidence interval, and bars depict the maximal and minimum range of values. Red-filled square: women (N = 8) and blue-filled circle: men (N = 12).

The τ_1off_ correlated, exceptionally, to BE-slope for the v200 m trial, with low level and unsatisfactory sample power, and the MRT_1off_ correlated to BE-slope for both v200 m and v90%Δ trials, but with low level and unsatisfactory sample power. The variability of $EE{\dot{V}O}_{2}$ (at IIST_v200m and v90%Δ) values is closer to that observed for ${\dot{V}O}_{2peak}$ values when compared to the variability observed for EE $\dot{V}$ O_2_ at v200 m and $BE-{\dot{V}O}_{2peak}$ estimates in all trials, with the largest shown in v200 m (Figure 4).

**FIGURE 5:**
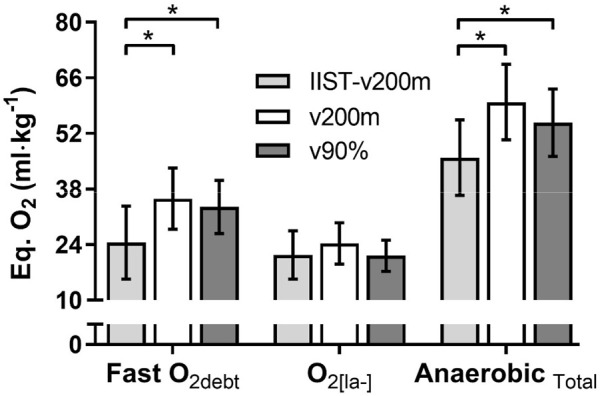
Anerobic energy demand during IIST_v200m, v200m, and v90%Δ trials: comparison between each trial regarding the responses of phosphagen (fast-O_2debt_), glycolytic (O_2[la^−^]_), and total anaerobic (Anaer_Total_).

The fast-O_2debt_, O_2[la^−^]_ and AnaerTotal demands assessed during the IIST_v200m, v200 m, and v90%Δ trials are shown in Figure 5. The fast-O_2debt_ post IIST_v200m was lower (*p* < 0.01, η^2^= 0.281, considered [medium] effect size) than those post v200 m and v90%Δ. However, the values of O_2[la^−^]_ were not different (*p* = 0.11) between IIST_v200m, v200 m, and v90%Δ. The Anaer_Total_ also was lower (*p* < 0.01, η^2^= 0.294, considered [medium] effect size) than those post v200m and v90%Δ. No correlations were observed between fast-O_2debt_ and O_2[la^−^]_ values with the responses of $EE{\dot{V}O}_{2}$ , $BE-{\dot{V}O}_{2peak}$ , and BE-slope for IIST_200m, v200 m, and v90%Δ, respectively. However, τ_1off_ and MRT_1off_ were moderately related to Anaer_Total_ post IIST_v200m (r = 0.64 and r = 0.66; *p* < 0.01), v200 m (r = 0.61 and r = 0.52; *p* < 0.01 and *p* = 0.02), and v90%Δ (r = 0.64 and r = 0.57; *p* < 0.01).

## Discussion

The assumption that maximal ${\dot{V}O}_{2}$ response (i.e., ${\dot{V}O}_{2peak}$ ) can be elicited, and therefore assessed, during the trials was evidenced from the comparison between mean values of ${\dot{V}O}_{2peak}$ , $EE{\dot{V}O}_{2}$ , and $BE-{\dot{V}O}_{2peak}$ . In contrast, whether ${\dot{V}O}_{2peak}$ can be assessed with reliability by BE procedures applied under different recovery conditions in the severe-intensity domain requires further considerations. For example, the estimated $BE-{\dot{V}O}_{2peak}$ showed low-to-moderate coefficients for the explained variance of the ${\dot{V}O}_{2peak}$ values assessed in the incremental test, with lowest coefficients observed for the 200-m single trial, which means that BE might mismatch actual ${\dot{V}O}_{2peak}$ between swimmers irrespective of the trial condition, but mainly in the 200-m trial. Also, when $BE-{\dot{V}O}_{2peak}$ is estimating $EE{\dot{V}O}_{2}$ , an improved coefficient of explanation is observed for single-trial conditions, which means that BE provides a satisfactory assessment of ${\dot{V}O}_{2}$ elevation during swimming in the severe-intensity domain. Moreover, the transients of ${\dot{V}O}_{2}$ off-kinetics played an important role on the reliability of $BE-{\dot{V}O}_{2peak}$ estimate since delayed and slowed time courses of ${\dot{V}O}_{2}$ recovery overshoot the BE values, which seemed to be a direct and positive effect of Anaer_Total_ release on the transients of ${\dot{V}O}_{2}$ off-kinetics.

First, it is important to note that linear fitting underlying the BE mathematical procedure showed high adjustment coefficients for the 20 s dataset (with fixed TD = 15 s), irrespective of the trial performance in the severe-intensity exercise domain. Hence, the current finding indicating possible mismatching between ${\dot{V}O}_{2peak}$ and $BE-{\dot{V}O}_{2peak}$ should not be addressed to the robustness (i.e., reduced regression power) of the linear procedure applied to the current estimates. The concerns when a fixed delay is considered in the initial phase of ${\dot{V}O}_{2}$ recovery are related to the accuracy of the estimate. Commonly, studies have demonstrated that the accuracy of the BE model is increased when selecting 20 s of data (Chaverri et al., 2016; Rodíguez et al., 2017; Monteiro et al., 2020), applying a linear fit strategy, and considering a short delay (e.g., ∼5–10 s) before dataset fitting, which is, however, not a consensus for BE estimates in different exercise domains (Monteiro et al., 2020) and the exertion level or performance condition at a given exercise domain (Chaverri et al., 2016; Rodíguez et al., 2017). The current finding did not disagree with the aforementioned recommendations for the application of BE procedures but instead suggested that such an arbitrary delay of 15 s shall ensure that the ${\dot{V}O}_{2}$ recovery post-swimming performance in severe-intensity domains has already been initiated, and, indeed, the linear fitting strategy on the 20-s dataset still presents high accuracy for the BE estimate.

Second, there is robust statistical evidence from the comparisons between mean values of ${\dot{V}O}_{2peak}$ and $BE-{\dot{V}O}_{2peak}$ that these measurements are interchangeable, irrespective of the trial in which the $BE-{\dot{V}O}_{2peak}$ was estimated. Similar evidence was also observed comparing mean values of EE $\dot{V}$ O_2_ and $BE-{\dot{V}O}_{2peak}$ . However, dispersion plots of ${\dot{V}O}_{2peak}$ vs. $BE-{\dot{V}O}_{2peak}$ refuted the interchangeable use between each other, showing that the power with which ${\dot{V}O}_{2peak}$ was estimated from $BE-{\dot{V}O}_{2peak}$ in the post IIST_v200m, v90%Δ, and v200 m trials attained, respectively, moderate (44 and 43%) or low (18%) rates, with just the first two rates with satisfactory statistical confidence. Therefore, the $BE-{\dot{V}O}_{2peak}$ post v200 m seems to be an unreliable assessment of ${\dot{V}O}_{2peak}$ , which might be attributed to the tendency (not significant) to overestimate actual values.

However, the ${\dot{V}O}_{2}$ final response during all trials (i.e., $EE{\dot{V}O}_{2}$ ) attained maximal rates, and hence it did not account for the mismatching between ${\dot{V}O}_{2peak}$ vs. $BE-{\dot{V}O}_{2peak}$ either post v200 m or post IIST_v200 m and v90%Δ. Indeed, the assumption that maximal ${\dot{V}O}_{2}$ response is elicited during a 200-m single-trial performance has been well-reported (Almeida et al., 2020; Sousa et al., 2011; Rodríguez et al., 2017) and thus also contributing to recognize no constraints to the attainment of ${\dot{V}O}_{2peak}$ in 200 m. Furthermore, the current and previous reports on ${\dot{V}O}_{2}$ response in 200 m also contribute to the typification of the severe-intensity domain in such distance and recognized for swimming conditions ranging from 95 to 105% of v $\dot{V}$ O_2max_ (Sousa et al., 2014), or even for swimming velocity corresponding to 70%Δ (Reis et al., 2012), and just above the respiratory compensation point (Pessoa-Filho et al., 2012).

Third, whether there are no mathematical or physiological concerns about the reliability of BE procedures after all trials, why were the estimates considered poor (and unsatisfactory) for v200 m and moderate (but satisfactory) for IIST_v200m and v90%Δ? The effect of the energetics components during trial performances on the ${\dot{V}O}_{2}$ initial recovery phase might provide new insights into the reliability of BE. Despite the lack of information regarding the effect of aerobic/anerobic energy release on ${\dot{V}O}_{2}$ off-kinetics post-swimming performance in the severe-intensity domain since previous studies just analyzed the ${\dot{V}O}_{2}$ recovery profile in response to exercises at or around maximal aerobic values (i.e., 100% or ranging from 95 to 105% ${\dot{V}O}_{2peak}$ , Sousa et al., 2014, 2015) or even at a given distance (i.e., 200 m; Sousa et al., 2011; Almeida et al., 2020), the current findings evidenced that total anerobic energy (i.e., phosphagenic in addition to glycolytic components) released during each trial showed a moderate and positive relationship with the transients τ_1off_ and MRT_1off_. This means that the trials demanding higher anerobic release might also be associated to slower ${\dot{V}O}_{2}$ recovery, as observed when comparing the slow responses post v90%Δ and v200 m with the fast post IIST_v200m.

In other sports than swimming, longer transients for ${\dot{V}O}_{2}$ off-kinetics were associated with different intramuscular mechanisms such as 1) the rate of phosphocreatine resynthesis (i.e., a higher amount of phosphocreatine to restore requires a longer ${\dot{V}O}_{2}$ decrement phase; Rossiter et al., 2002; Korzeniewski and Zoladz, 2013); 2) lactate clearance (i.e., parallel lactate oxidation and transportation slow the time course of ${\dot{V}O}_{2}$ recovery; (Cunningham et al., 2000;; Özyener et al., 2001); and 3) the pattern of type II fiber recruitment (i.e., the inefficiency of oxidative phosphorylation also accounts to increase the time course of ${\dot{V}O}_{2}$ recovery (Cunningham et al., 2000; Rossiter et al., 2002).

Particularly, in swimming, longer ${\dot{V}O}_{2}$ time course during recovery has also been reported after the trial (200 m) and time-limited performance (Sousa et al., 2011, 2015), which was attributed to both the slower ${\dot{V}O}_{2}$ response until maximal values and to the accumulation of fatigue-related metabolites while performing each swimming condition. Although the current study has no information on the time course of ${\dot{V}O}_{2}$ on-kinetics response, which is therefore a limitation to be more assertive regarding the symmetry between on- and off-transients of ${\dot{V}O}_{2}$ response, the current findings are best aligned with the statement that a longer ${\dot{V}O}_{2}$ decrease is also probably linked to the anerobic reliance during swimming performance in the severe-intensity domain.

Moreover, the EE $\dot{V}$ O_2_ did not differ between IIST_v200m, v200m, and v90%Δ, and no differences were observed for A_1off_ after each trial. In cycling, the similarity of ${\dot{V}O}_{2}$ values and ${\dot{V}O}_{2}$ on-kinetics between different performances in high-intensity exercise is consistent with the assumption that the attainment of a maximal oxidative response is not affected by the pattern of fast/slow fiber type recruitment, and its particular metabolic profile for each trial, i.e., cost of O_2_, rate of phosphate utilization, amplitude of slow component, and accumulation of metabolites (Cunninghan et al., 2000; Özyener et al., 2001; Rossiter et al., 2002). Therefore, there are also no physiological arguments to suppose that ${\dot{V}O}_{2peak}$ was not attained while performing v200m, IIST_v200m, and v90%Δ, even considering that differences were observed between them regarding total anerobic demand.

However, the aforementioned metabolic statement in cycling also inferred that longer transients of the initial ${\dot{V}O}_{2}$ recovery phase are probably related to the reliance on type II fibers during the performance in the severe-intensity domain, as suggested by higher anerobic release and slow component occurrence, respectively, for higher-intensity short trials (i.e., fast fiber contribution is promptly established) and longer-term trials (i.e., fast fiber contribution is progressively established) (Cunninghan et al., 2000; Özyener et al., 2001; Rossiter et al., 2002). While the current finding on the positive correlation between A_1off_ with $BE-{\dot{V}O}_{2peak}$ and $EE{\dot{V}O}_{2}$ in all trials is aligned with the symmetry between the amplitude of ${\dot{V}O}_{2}$ recovery and its values attained during exercise, the positive correlation in all trials between total anerobic energy and MRT (even if in the moderate level) is also consistent with the muscular bioenergetics (with high reliance on anaerobic energy) having influence on ${\dot{V}O}_{2}$ recovery time course, which therefore accounted for the observation of MRT relationship to BE-slope only in v200 m and v90%Δ.

Finally, the findings suggested that the initial amplitude of ${\dot{V}O}_{2}$ off-kinetics does not account for the possible mismatch between ${\dot{V}O}_{2peak}$ and $BE-{\dot{V}O}_{2peak}$ , unless the attained value of $EE{\dot{V}O}_{2}$ is lower than that of ${\dot{V}O}_{2peak}$ (i.e., therefore the assumption of maximal ${\dot{V}O}_{2}$ cannot be ensured). Moreover, the anerobic energy released contributes moderately to the longer transients of ${\dot{V}O}_{2}$ off-kinetics, which suggests that the muscular metabolism is one among other variables with effect on $BE-{\dot{V}O}_{2peak}$ reliability. However, the current results cannot address the reasons underpinning the better matching between ${\dot{V}O}_{2peak}$ and $BE-{\dot{V}O}_{2peak}$ in v90%Δ than in v200 m. Although the aerobic contribution to each trial (i.e., total demand of ${\dot{V}O}_{2}$ ) was not measured in the current study, it is expected to be higher in v90%Δ than in v200 m as supported when comparing previous reports on the energetics for swimming at velocities surrounding maximal aerobic velocity (Sousa et al., 2014) or at 200 m (Massini et al., 2021).

From the results of these previous studies, the reliance on oxidative metabolism during the performance of v90%Δ is supposed to be higher than that of v200 m, and thus the attainment of a given value of EE $\dot{V}$ O_2_ not different from $EE{\dot{V}O}_{2}$ not different from ${\dot{V}O}_{2peak}$ is expected for each swimmer and can be accounted to the low variability of $BE-{\dot{V}O}_{2peak}$ estimate during v90%Δ. Therefore, the lack of information on aerobic contribution is another limitation of the current study, which should be overcome in future studies aiming to address whether the muscular energetics influence ${\dot{V}O}_{2}$ on-kinetics when comparing distance-limited and time-limited performances in swimming. It can be argued that the poor matching between ${\dot{V}O}_{2peak}$ and $BE-{\dot{V}O}_{2peak}$ in v200 m is a feature of the fixed delay (15 s) applied to the BE procedure. Despite the reliability of the ${\dot{V}O}_{2peak}$ estimate being susceptible to different time delays (Rodígues et al., 2017; Monteiro et al., 2020), the initial ${\dot{V}O}_{2}$ recovery seems to differ from 15 s only for IIST_v200m, in which the $BE-\dot{V}O_{2peak}$ estimate was not suspicious.

Although the scope of the current study was not the analysis of the effect of data treatment on the measurements of the transients and amplitudes of ${\dot{V}O}_{2}$ kinetics and BE, an unstudied issue in swimming physiology is whether breathing mechanics (i.e., ventilatory frequency and volume) is disturbed with the AquaTrainer^®^ apparatus by comparing to actual free-swimming condition (e.g., producing larger set of aberrant ${\dot{V}O}_{2}$ data). It is important to investigate whether swimming has an intrinsic characteristic of ventilatory mechanics, which is different from other sports, hence requiring proper ${\dot{V}O}_{2}$ dataset treatment.

When analyzing the practical applications of the current findings, three major comments are discernible: 1) BE is a feasible procedure for the assessment of ${\dot{V}O}_{2}$ response at the end of exercise conditions in the severe-intensity domain (represented by IIST_v200m, v200m, and v90%Δ in the current study), which approached a maximal aerobic value despite the lack of endorsement on its interchangeability with ${\dot{V}O}_{2peak}$ ; 2) such a maximal aerobic value is, however, meaningful for coaches as it represents the muscular oxidative profile in the severe-intensity domain, and hence enabling the management of aerobic response in middle-distance performance, the adjustments with cardiorespiratory conditioning during training demanding maximal aerobic responses, and the pace reference for training in the severe-intensity domain; and 3) the BE protocol with best reliability to assess the ${\dot{V}O}_{2}$ response that matches ${\dot{V}O}_{2peak}$ should allow a proportionally higher reliance on aerobic than anerobic energy contribution, as is probably the case either during longer trials in the severe-intensity domain (e.g., 300–400 m) or shorter distances preceding a similar trial (e.g., 2 × 200 m).

## Conclusion

The major contribution of the current study was to determine the effect of anerobic response on the reliability of the estimation of ${\dot{V}O}_{2peak}$ by BE, demonstrating that the anerobic demand might also be associated to longer transients of ${\dot{V}O}_{2}$ off-kinetics (i.e., slowed ${\dot{V}O}_{2}$ recovery), which in turn are associated to the alterations of the slope of the regression line (e.g., reducing the inclination), and therefore compromising the reliability of ${\dot{V}O}_{2peak}$ estimate, in spite of the strength of these associations observed to be low to moderate. Considering the fact that performance in a single effort with significant contribution of anerobic energy (as observed for v200m and v90%Δ) should probably demand a significant time constant or average response time of ${\dot{V}O}_{2}$ recovery; a useful solution is to ensure faster responses of the transients of ${\dot{V}O}_{2}$ off-kinetics, with the performance of an exercise with the same characteristics of effort intensity as the one where the test is intended to be carried out, as observed in the ISST_v200m situation. In addition, the findings also reinforce that the time delay for ${\dot{V}O}_{2}$ recovery should be considered to apply BE procedures in trials in the severe-intensity domain, being recommendable to encompass a dataset no larger than 15 s. Finally, another important piece of evidence is the response of ${\dot{V}O}_{2}$ at the end of IIST_v200m, v200 m, and v90%Δ corresponding to that typical of the severe-intensity domain, despite the estimation of ${\dot{V}O}_{2peak}$ by BE giving no confident value from the v200m test, and hence the estimates from IIST_v200m and v90%Δ are preferable for planning trials, controlling oxidative response, and monitoring the conditioning adjustment needed to perform in the severe-intensity domain.

## Data Availability

The raw data supporting the conclusions of this article will be made available by the authors, without undue reservation.
